# Left and Right Ventricular Hemodynamic Response After Transcatheter Mitral Valve Replacement

**DOI:** 10.1016/j.shj.2024.100322

**Published:** 2024-06-08

**Authors:** Sebastian Ludwig, Lena S. Strotmann, Benedikt N. Schrage, Benedikt Koell, Augustin Coisne, Andrea Scotti, Karl-Philipp Rommel, Jessica Weimann, Michael Schwarzl, Moritz Seiffert, Hermann Reichenspurner, Stefan Blankenberg, Andreas Schäfer, Daniel Burkhoff, Niklas Schofer, Juan Granada, Lenard Conradi, Daniel Kalbacher

**Affiliations:** a Department of Cardiology, University Heart & Vascular Center Hamburg, University Medical Center Hamburg-Eppendorf, Hamburg, Germany; b German Center for Cardiovascular Research (DZHK), Hamburg, Germany; c Cardiovascular Research Foundation, New York, New York; d Univ. Lille, Inserm, CHU Lille, Institut Pasteur de Lille, Lille, France; e Montefiore-Einstein Center for Heart and Vascular Care, Montefiore Medical Center, New York, New York; f Department of Internal Medicine/Cardiology, Leipzig University, Heart Center, Leipzig, Germany; g Abteilung für Innere Medizin, Bezirkskrankenhaus Schwaz, Schwaz, Austria; h Department of Cardiovascular Surgery, University Heart & Vascular Center Hamburg, University Medical Center Hamburg-Eppendorf, Hamburg, Germany

**Keywords:** Hemodynamics, Mitral regurgitation, Pressure-volume relationship, Transcatheter mitral valve replacement

## Abstract

**Background:**

Transcatheter mitral valve replacement (TMVR) represents a novel treatment option for patients with mitral regurgitation (MR), but little is known about the hemodynamic impact of MR elimination following TMVR. We sought to investigate the hemodynamic impact of TMVR on left ventricular (LV) and right ventricular (RV) function using noninvasive pressure-volume loops.

**Methods:**

All consecutive patients undergoing TMVR with dedicated devices between May 2016 and August 2022 were enrolled. The end-diastolic and end-systolic pressure-volume relationships were estimated from 26 patients using single-beat echocardiographic measurements at baseline and after TMVR at discharge. RV function was assessed by RV-pulmonary artery (PA) coupling and RV fractional area change. One-year follow-up was available for 19 patients. The prognostic impact of calculated end-diastolic volume at an end-diastolic pressure of 20 mmHg (VPed20) reduction was assessed by Cox regression.

**Results:**

A total of 26 patients (77.0 years [interquartile range 73.9-80.1], N = 17 [65.4%] male) with successful TMVR were included (secondary MR [N = 21, 80.8%]; median LV ejection fraction was 37.0% [interquartile range 30.7-50.7]). At discharge, a decrease in VPed20 (*p* < 0.001) indicating leftward shift of end-diastolic pressure-volume relationship, and an increase of the end-systolic elastance slope (*p* = 0.007) were observed after TMVR. No changes were observed for RV-PA coupling (*p* = 0.19) and RV fractional area change (*p* = 0.22). At 1-year follow-up, LV contractility (end-systolic elastance) and RV-PA coupling remained stable. Vped20 reduction at discharge was significantly associated with 1-year all-cause mortality or heart failure hospitalization (hazard ratio 0.16, 95% CI 0.04-0.71, *p* = 0.016).

**Conclusions:**

Noninvasive assessment of pressure-volume loops demonstrated early LV reverse remodeling and improved LV contractility, while RV performance was preserved. These results indicate the potential prognostic impact of complete MR elimination after TMVR.

## Graphical abstract

**Figure:**
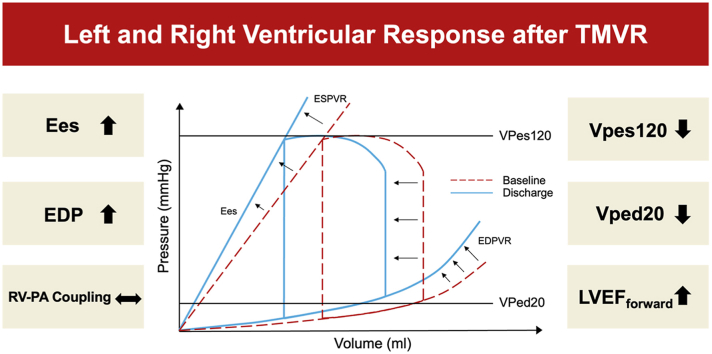

## Introduction

Mitral regurgitation (MR) is the most common valvular heart disease in industrialized countries with an increasing incidence due to an aging society.^1^ If left untreated, MR can lead to elevated left ventricular (LV) preload, volume overload, and eventually heart failure (HF) contributing to increased mortality rates.^2^ Patients with severe symptomatic MR should be treated surgically (i.e., mitral valve repair or replacement) or by transcatheter edge-to-edge repair (TEER) according to international guidelines.^3^^,^^4^ However, there is a significant subset of patients who are either ineligible for surgery because of comorbidities or advanced age or who present unfavorable anatomy for TEER. For these patients, transcatheter mitral valve replacement (TMVR) represents a potential alternative. Early clinical experience with different TMVR devices has demonstrated effective and durable MR elimination in the vast majority of patients treated.^5^^,^^6^ While residual and recurrent MR occur frequently in patients undergoing TEER, MR elimination is considered the central therapeutic advantage of TMVR over TEER.^7^^,^^8^ Although it is well known that residual MR after TEER can have adverse effects and that complete MR elimination through TMVR seems beneficial in theory, the implications of complete MR elimination on ventricular performance and prognosis in TMVR patients remain uncertain and have only been investigated sporadically before.^8^, ^9^, ^10^, ^11^, ^12^

Therefore, we sought to investigate the effect of MR elimination through TMVR on both LV hemodynamic and volumetric changes, as well as right ventricular (RV) function, in patients with severe MR using noninvasive LV pressure-volume loops derived from single-beat echocardiographic measurements. By analyzing LV hemodynamic and volumetric changes, as well as RV function, we aim to gain a better understanding of the implications of MR elimination through TMVR on ventricular performance and prognosis in this patient population.

## Methods

### Study Design

**Figure 1:**
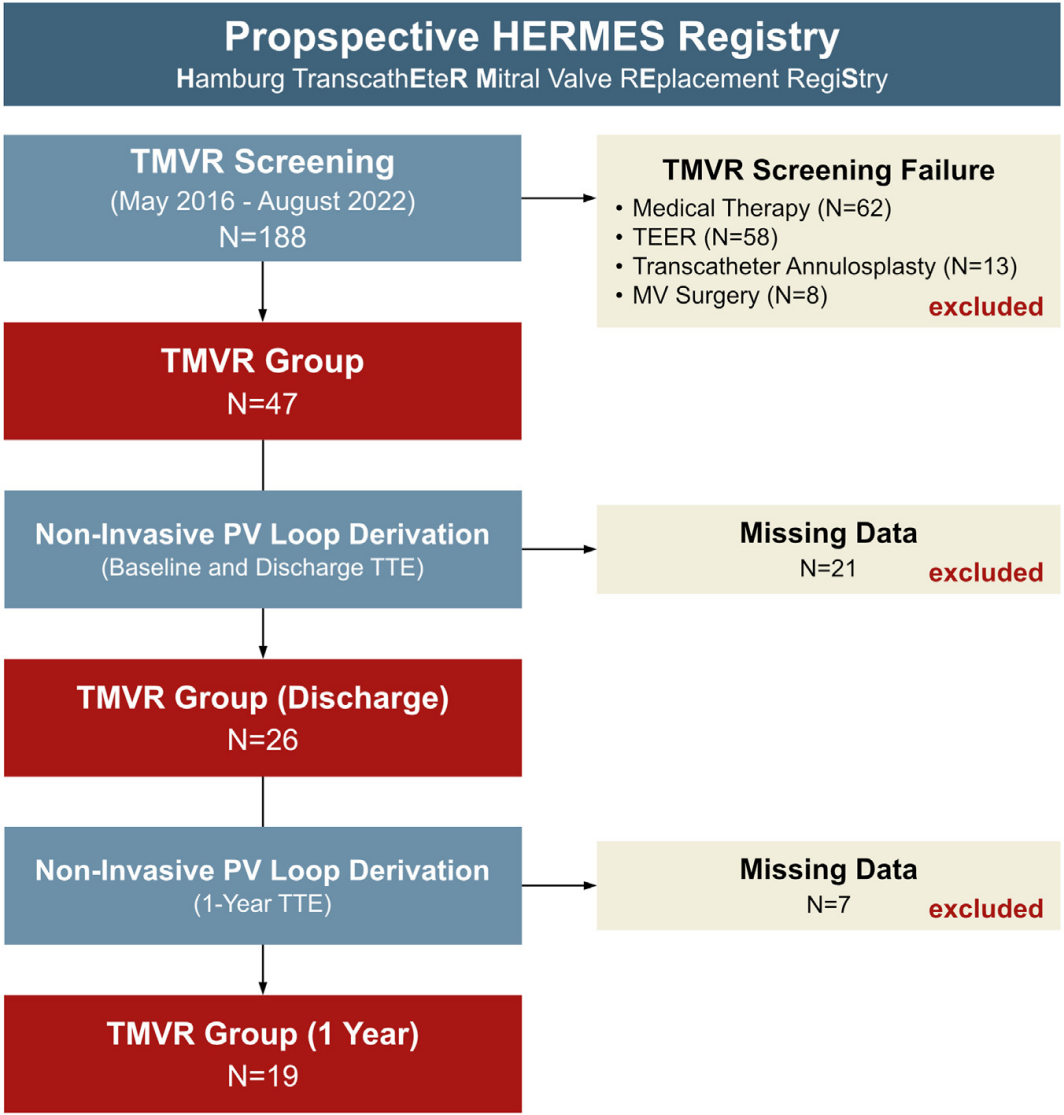
**Study flow chart** Abbreviations: MV, mitral valve; PV, pressure volume; TEER, transcatheter edge-to-edge repair; TMVR, transcatheter mitral valve replacement; TTE, transthoracic echocardiograph.

Data for the conduct of this study were derived from the HERMES prospective, observational, single-center registry (*Hamburg TranscathEteR Mitral Valve Replacement RegiStry*; ClinicalTrials.gov: NCT04914468). In total, this registry included 188 patients with clinically significant mitral valve disease undergoing screening for TMVR devices from May 2016 to August 2022, according to an interdisciplinary screening process described before.^13^^,^^14^ All patients were considered to have complex, nonfavorable TEER anatomy and were at high or prohibitive surgical risk. Among these, a total of 47 patients underwent TMVR using dedicated transapical or transfemoral/transseptal devices. Implanted TMVR devices included Tiara [Neovasc], Tendyne [Abbott Vascular], HighLife [HighLife Medical], or CardiAQ [Edwards Lifesciences] valves. Each case was evaluated by an interdisciplinary heart team, and decisions were based on good clinical standards. For this study, 26 consecutive patients undergoing TMVR with available single-beat echocardiographic data to derive noninvasive pressure-volume loops at baseline and discharge were included. Discharge echocardiography was performed at a mean of 14 days after the intervention. One-year follow-up was available in 19 patients and was obtained after 12 ± 4 months. One patient was treated via transfemoral/transseptal approach, and 25 patients were treated via transapical approach (Figure 1).

### Echocardiographic Measurements

All echocardiographic measurements were performed by experienced physicians based on current recommendations as part of the standard clinical work-up in the echocardiography laboratory of the structural heart disease department.^15^ Forward stroke volume (SV_forward_) was determined from the product of the LV outflow tract diameter and the LV outflow tract velocity time integral (measured by pulsed-wave Doppler). Left ventricular ejection fraction (LVEF) was calculated using the Simpson method, and forward LVEF (LVEF_forward_) was measured as SV_forward_ divided by the end-diastolic volume (EDV). Early diastolic tissue velocity was determined by pulsed-wave tissue Doppler imaging, assessing the septal and lateral mitral annular plane velocity and taking the mean of both measurements. Early trans-mitral diastolic peak velocity was measured by pulsed-waved Doppler imaging by placing the sample volume at the tip of the mitral leaflets at baseline and at the valve plane after valve implantation. In patients with atrial fibrillation, sequential measurements were performed and mean values were calculated.

RV function was determined by measurement of tricuspid annular plane systolic excursion (TAPSE), pulmonary artery systolic pressure (PASP), RV-pulmonary artery (RV-PA) coupling, as well as RV fractional area change (RVFAC).^16^ Based on apical four-chamber views, TAPSE was measured by placing an M-mode line at the lateral tricuspid valve annulus. The tricuspid regurgitant jet was assessed by a continuous-wave Doppler placed to obtain the maximum velocity. Based on the Bernoulli equation, the maximum velocity was transferred into pressure values. The peak value plus the estimated right atrial pressure was taken as the PASP. The RV-PA coupling was defined as the TAPSE/PASP ratio. RVFAC was obtained by tracing the RV endocardial border at end-diastole as well as end-systole in apical four-chamber views.

### Noninvasive Hemodynamic Measurements

End-diastolic and end-systolic pressure-volume relationships (EDPVR and ESPVR) were derived from estimating their positions using echocardiographic data in a single-beat method as previously described.^17^, ^18^, ^19^ According to this method, the equation end-diastolic pressure [EDP] = α EDV^β^ describes the EDPVR, with EDP defined as the end-diastolic pressure and EDV as the end-diastolic volume. The variables α and β are calculated as curve-fit parameters. With the assumption of a common underlying shape for volume-normalized EDPVRs, the curve-fit parameters α and β can be obtained for each individual patient from a single EDV/EDP data set.^18^ EDP was assessed using the equation EDP = 11.96+ (0.596 x early mitral inflow velocity/early diastolic tissue velocity).^20^ To obtain an estimate of the entire EDPVR and to compare its change after intervention, we used the calculated EDV at an EDP of 20 mmHg, known as VPed20.^18^^,^^21^

For the estimation of ESPVR and end-systolic elastance (Ees), respectively, we used a method assuming a linear relation of ESPVR as published by Chen et al.^19^ To describe the Ees noninvasively, we assessed systolic and diastolic brachial artery cuff pressures, forward stroke volume, and an estimated normalized ventricular elastance at arterial end-diastole. To estimate end-diastole, group-averaged values were used and adjusted using ejection fraction, systolic and diastolic arterial blood pressure, pre-ejection time, and total ejection time. Pre-ejection and total ejection time were derived from the LV outflow tract pulsed-wave Doppler. Ees, echocardiography derived end-systolic volume (ESV), and systolic blood cuff pressures were used for calculation of the x-axis intersection of the linear ESPVR (V0). In order to compare the position of the ESPVR with integration of V0 and Ees, we calculated ESV at an end-systolic pressure (ESP) of 120 mmHg, known as the systolic parameter VPes120.^19^ Ea describes the effective arterial elastance, which is derived from a 3-element Windkessel model described by Kelly et al.^22^^,^^23^ Ea can be determined approximately by using the arterial ESP and stroke volume.^24^ The ratio of Ea/Ees indicates the interaction between cardiac contractility and the arterial system.^25^ Systemic vascular resistance was assessed through pulsed-wave Doppler imaging as the ratio of peak mitral regurgitant velocity and the LV outflow time-velocity integral.^26^ These methods were then used to compare the hemodynamic status between baseline and discharge, and between discharge and 1-year follow-up (in patients with available data).

### Statistical Analysis

Continuous variables are shown as medians (25th percentile and 75th percentile) or as means ± standard deviation. Binary variables are described as counts (frequencies). To assess differences before and after TMVR, paired Student's t-test is applied. A *p* value of <0.05 was considered statistically significant. Cox regression for the combined 1-year endpoints of all-cause mortality or HF hospitalization and cardiovascular mortality or HF hospitalization were performed. Combining several potential risk factors the adjustment model included only EuroSCORE II due to the small sample size of this study. All analyses were performed with R statistical software version 4.0.3 (R Foundation for Statistical Computing, Vienna, Austria).

## Results

### Clinical and Echocardiographic Baseline Characteristics

A detailed description of the baseline variables is given in Table 1. A total of 26 patients including 17 male and 9 female patients were enrolled in the present study. Median age of the group was 77 years (interquartile range [IQR] 73.9-80.1), with a median body mass index of 27 kg/m^2^ (IQR 24.9-30.9). All patients were symptomatic according to the New York Heart Association functional class (New York Heart Association II: N = 2 [7.7%]; III: 20 [76.9%]; IV: 4 [15.4%]) and exhibited an elevated surgical risk according to the calculated EuroSCORE II (median: 5.5%; IQR 2.6-17.2). Two patients (7.7%) presented with chronic obstructive pulmonary disease and 17 patients (65.4%) suffered from atrial fibrillation. Baseline median estimated glomerular filtration rate was 39 mL/min/1.73 m^2^ (IQR 31.3-60.0). Previous myocardial infarctions occurred in eight patients (30.8%). Ten patients (38.5%) underwent previous cardiac surgery, of whom seven (26.9%) received coronary artery bypass graft surgery. All patients presented with MR 3+ (N = 3, 11.5%) or 4+ (N = 23, 88.5%). Secondary MR etiology was predominant (N = 21, 80.8%). Baseline LVEF was 39.2% ± 13.2% with a mean EDV of 167.8 ± 77.3 mL. RV parameters showed overall elevated PASP (50.5 ± 16.5 mmHg) and a RV-PA coupling ratio of 0.41 ± 0.21 mm/mmHg.

**Table 1:** Clinical baseline characteristics of the study population

Clinical baseline characteristics (N = 26)	
Age, y	77.0 (73.9, 80.1)
Sex, male	17 (65.4%)
BMI, kg/m^2^	27.0 (24.9, 30.9)
EuroScore II, %	5.5 (2.6, 17.2)
Diabetes mellitus	6 (22.8%)
COPD	2 (7.7%)
Hypertension	18 (69.2%)
Atrial fibrillation	10 (38.5%)
CAD	16 (61.5%)
Prior myocardial infarction	8 (30.8%)
eGFR, mL/min/1.73 m^2^	39.0 (31.3, 60.0)
Previous cardiac surgery	10 (38.5%)
Previous CABG	7 (26.9%)
NYHA functional class	
I	0
II	2 (7.7%)
III	20 (76.9%)
IV	4 (15.4%)
NT-proBNP, ng/L	4011.0 (2138.0, 5669.0)
Echocardiographic baseline characteristics	
MR etiology	
Primary MR	5 (19.2%)
Secondary MR	21 (80.8%)
EROA, cm^2^	0.3 (0.2, 0.4)
Regurgitant volume, mL	41.5 (30.5, 65.6)
LVEF, %	37.0 (30.7, 50.7)
Tricuspid regurgitation (≥moderate)	12 (46.2%)

Values are presented as median (IQR) or in percent (%).

Abbreviations: BMI, body mass index; CABG, coronary artery bypass graft; CAD, coronary artery disease; COPD, chronic obstructive pulmonary disease; eGFR, estimated glomerular filtration rate; EROA, effective regurgitant orifice area; LVEF, left ventricular ejection fraction; MR, mitral regurgitation, NT-proBNP, N-terminal pro b-type natriuretic peptide; NYHA, New York Heart Association.

### Echocardiographic Results

Following TMVR, MR was reduced to ≤1+ in all patients (N = 26, 100%). LV volumes in both end-diastole and end-systole (EDV and ESV) significantly decreased at discharge (EDV: 167.8 ± 77.3 mL vs. 139.0 ± 67.6 mL, *p* = 0.001; ESV: 113.4 ± 77.4 mL vs. 80.9 ± 73.7 mL, *p* < 0.001). In contrast, between discharge and 1-year follow-up, an increase of EDV and ESV was noted (EDV: 148.5.8 ± 68.3 mL vs. 174.2 ± 90.8 mL, *p* = 0.004; ESV: 92.7 ± 76.9 mL vs. 116.2 ± 89.7 mL, *p* = 0.053). LVEF remained stable from baseline to discharge (39% ± 13% vs. 36% ± 11%, *p* = 0.17), and from discharge to 1-year follow-up (36% ± 12% vs. 37% ± 12%, *p* = 0.64). Similarly, no changes were observed for SV_forward_ between baseline and discharge (54.4 ± 20.0 mL vs. 58.1 ± 18.8 mL, *p* = 0.42), as well as discharge and 1-year follow-up (55.8 ± 17.8 mL vs. 58.0 ± 20.9 mL, *p* = 0.70). LVEF_forward_ showed a significant increase from baseline to discharge (39% ± 22% vs. 53% ± 30%, *p* < 0.001) and remained stable thereafter (48% ± 28% vs. 44% ± 31%, *p* = 0.46). E/é increased significantly at discharge (17.9 ± 6.6 vs. 23.7 ± 8.6, *p* = 0.012) and did not change between discharge and 1-year follow-up (23.5 ± 9.1 vs. 25.0 ± 8.0, *p* = 0.57).

### Noninvasive Pressure-Volume Loops

**Figure 2:**
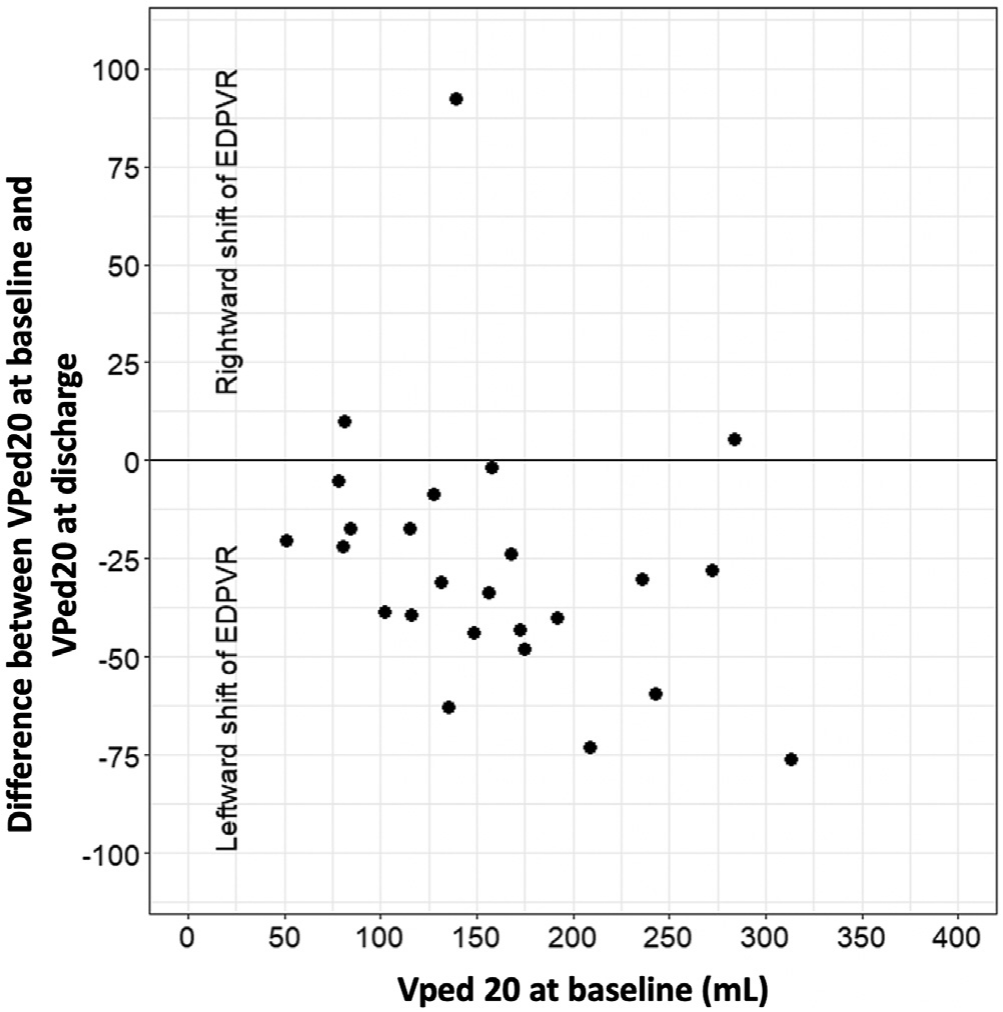
**End-diastolic pressure-volume relationship following TMVR.** Patients are compared based on their VPed20 as a marker of the EDPVR. The y-axis displays the change in VPed20 from baseline to discharge, and the x-axis displays the value of VPed20 at baseline Abbreviations: EDPVR, end-diastolic pressure-volume relationship; TMVR, transcatheter mitral valve replacement.; VPed20, calculated end-diastolic volume at an end-diastolic pressure of 20 mm Hg.

**Figure 3:**
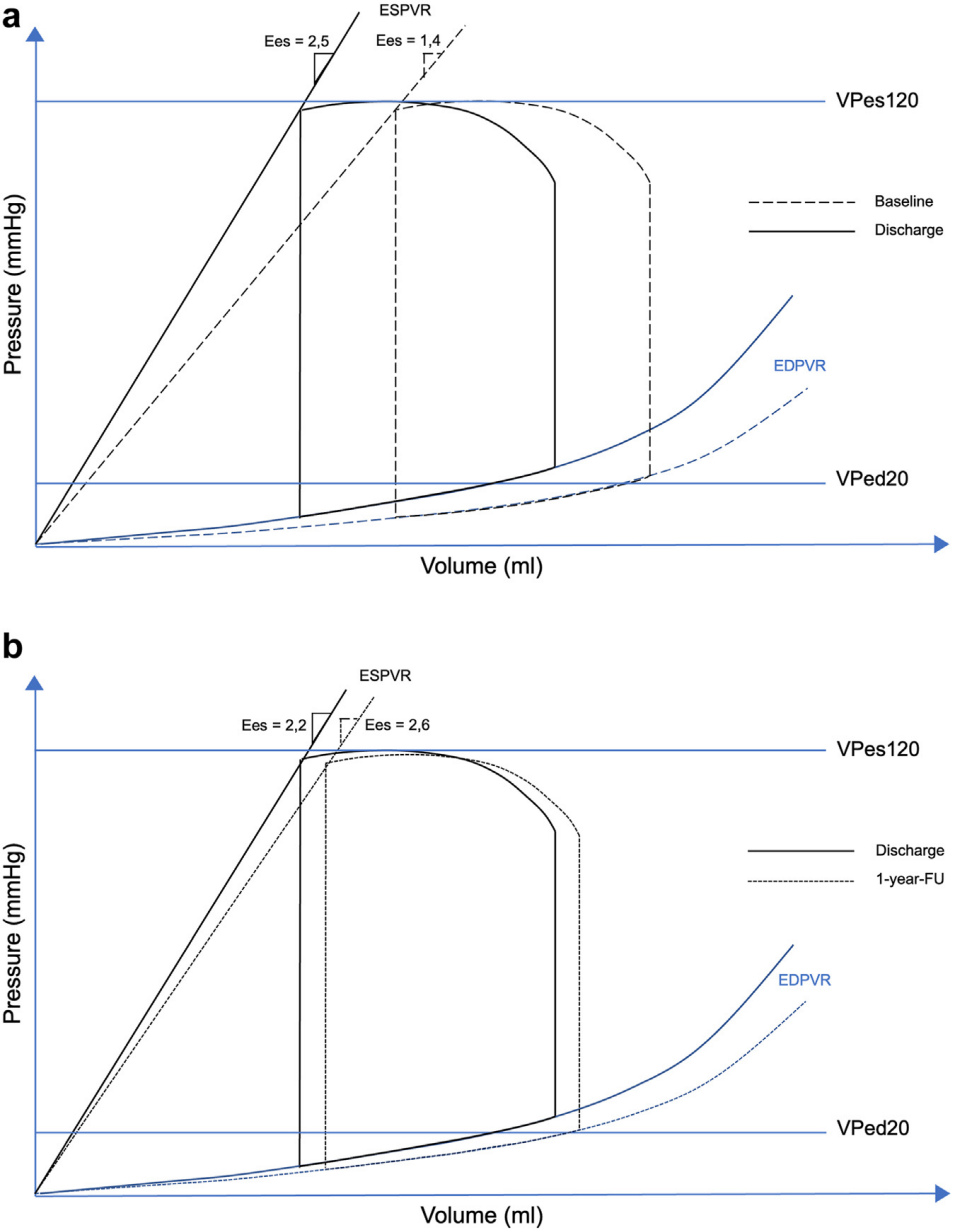
Schematic left ventricular pressure-volume visualization between (a) baseline and discharge and (b) discharge and 1-year follow-up. The ESPVR and EDPVR both shift leftward from baseline to follow-up and remain statistically unchanged from discharge to 1-year follow-up, despite an overall increase in LV volumes. VPed20 indicates an end-diastolic volume at a pressure of 20 mmHg, VPes120 indicates an end-systolic volume at a pressure of 120 mmHg, respectively. Ees indicates the slope of ESPVR Abbreviations: 1-year FU, 1-year follow-up; EDPVR, end-diastolic pressure-volume relationship; Ees, end-systolic elastance; ESPVR, end-systolic pressure-volume relationship.

**Figure 4:**
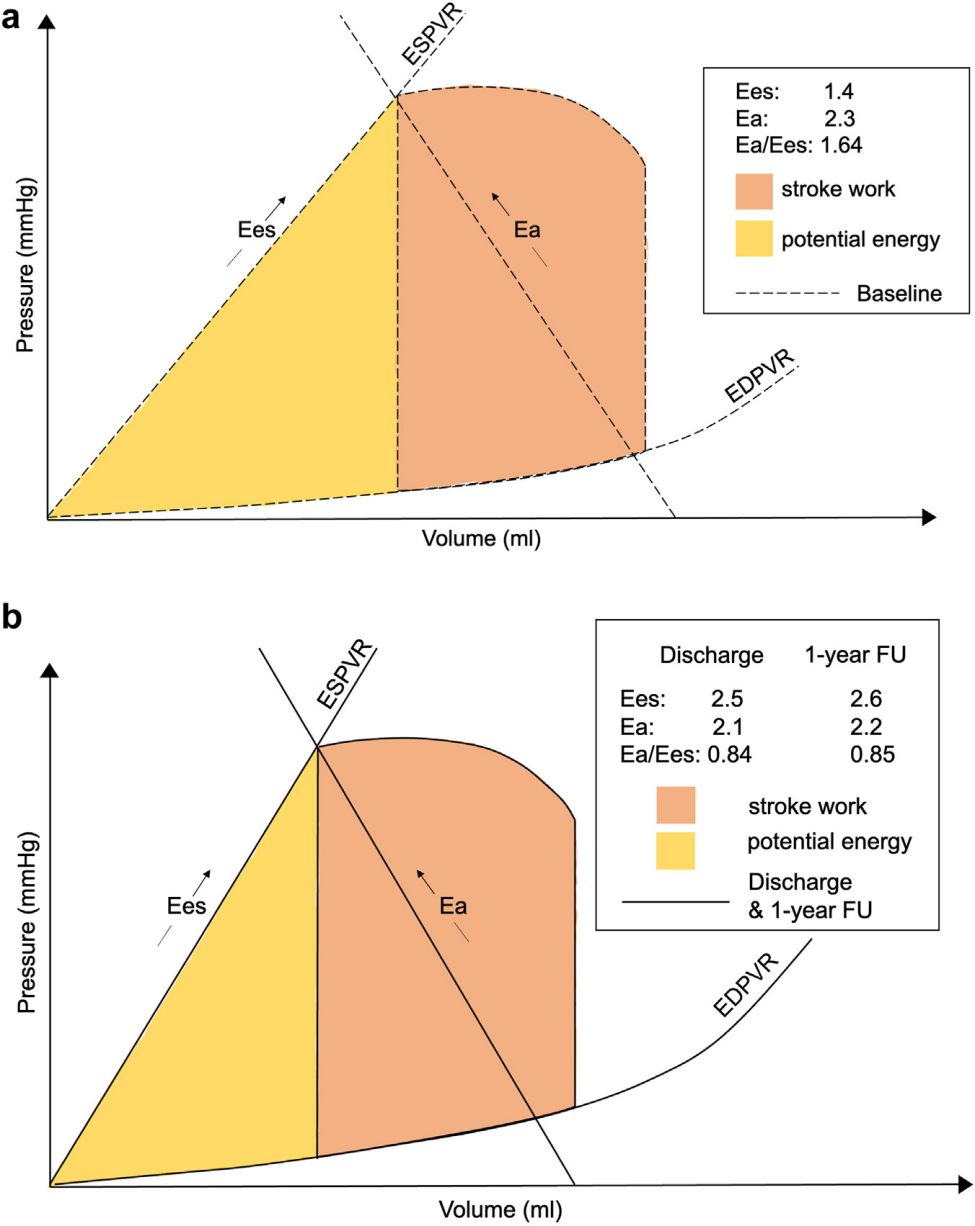
The PVA of a single beat at (a) baseline and (b) discharge and 1-year follow-up. The PVA can be divided into two parts: 1. The stroke work, which includes the area within the pressure-volume loop (orange). 2. The potential energy, which describes the triangular area formed by the ESPVR and EDPVR slopes (yellow) Abbreviations: 1-year FU, 1-year follow-up; Ea, effective arterial elastance; EDPVR, end-diastolic pressure-volume relationship; Ees, end-systolic elastance; ESPVR, end-systolic pressure-volume relationship; PVA, pressure-volume area.

A detailed description of hemodynamic changes following TMVR (baseline vs. discharge, discharge vs. 1-year follow-up) is given in Table 2. Comparing noninvasive pressure-volume loop results before and after TMVR, a significant decrease of VPed20 (165.3 ± 75.8 mL vs. 134.0 ± 65.9 mL, *p* < 0.001) was observed between baseline and discharge, indicating a leftward shift of the EDPVR toward overall lower volumes in most patients (Figure 2). Accordingly, the systolic parameter VPes120 (129.9 ± 94.4 mL vs. 91.4 ± 84.0 mL, *p* < 0.001) also decreased. At 1-year follow-up, VPed20 showed a statistically not nonsignificant trend toward an increase (142.9 ± 65.9 mL vs. 166.6 ± 88.1 mL, *p* = 0.052), whereas VPes120 remained stable (106.0 ± 89.1 mL vs. 135.5 ± 116.0 mL, *p* = 0.10) (Supplementary Figure 1). End-systolic elastance (Ees) showed a significant increase at discharge following TMVR (1.4 ± 0.9 vs. 2.5 ± 2.4, *p* = 0.007) and remained stable between discharge and follow-up (2.2 ± 2.4 vs. 2.6 ± 3.5, *p* = 0.60). This reflects an increase in the slope of ESPVR, which is displayed in the pressure-volume diagram (Figure 3). V0 did not change significantly from baseline to discharge (-3.1 ± 65.1 mL vs. -1.7 ± 38.3 mL, *p* = 0.90). A significant increase in EDP was observed following TMVR at discharge (22.6 ± 4.0 mmHg vs. 26.1 ± 5.1 mmHg, *p* = 0.012), which remained stable thereafter (26.0 ± 5.4 mmHg vs. 26.9 ± 4.8 mmHg, *p* = 0.57). In contrast, ESP did not show significant changes between baseline and discharge (110.6 ± 18.4 mmHg vs. 109.6 ± 13.8 mmHg, *p* = 0.77) and between discharge and 1-year follow-up (108.5 ± 14.2 mmHg vs. 109.7 ± 24.2 mmHg, *p* = 0.84). No significant changes in Ea and systemic vascular resistance were observed following TMVR. The Ea/Ees ratio declined from 1.64 at baseline to 0.84 at discharge and remained stable (Ea/Ees 0.85) between discharge and 1-year follow-up (Figure 4).

**Table 2:** Hemodynamic outcome data before and after TMVR

Variables	Baseline (N = 26)	Discharge (N = 26)	*P* value	Discharge (N = 19)	1-year follow-up (N = 19)	*P* value
Heart rate, bpm	74.0 ± 14.9	72.1 ± 15.4	0.56	72.1 ± 15.7	78.6 ± 21.7	0.15
ß	6.2 (6.1, 6.9)	6.4 (5.1, 7.5)	0.92	6.3 (5.1,6.6)	6.7 (5.1, 8.4)	0.21
Vped20, mL	165.3 ± 75.8	134.0 ± 65.9	<0.001	142.9 ± 65.9	166.6 ± 88.1	0.052
EDV, mL	167.8 ± 77.3	139.0 ± 67.6	0.0013	148.5 ± 68.3	174.2 ± 90.8	0.035
EDP, mmHg	22.6 ± 4.0	26.1 ± 5.1	0.012	26.0 ± 5.4	26.9 ± 4.8	0.57
E/é	17.9 ± 6.6	23.7 ± 8.6	0.012	23.5 ± 9.1	25.0 ± 8.0	0.57
é	0.0742 ± 0.0216	0.0587 ± 0.0191	0.011	0.0571 ± 0.0209	0.0553 ± 0.0217	0.76
Ea	2.3 ± 0.8	2.1 ± 0.6	0.27	2.1 ± 0.6	2.2 ± 1.0	0.80
ESV, mL	113.4 ± 77.4	80.9 ± 73.7	<0.001	92.7 ± 76.9	116.2 ± 89.7	0.053
ESP, mmHg	110.6 ± 18.4	109.6 ± 13.8	0.77	108.5 ± 14.2	109.7 ± 24.2	0.84
VPes120, mL	129.9 ± 94.4	91.4 ± 84.0	<0.001	106.0 ± 89.1	135.5 ± 116.0	0.10
Ees	1.4 ± 0.9	2.5 ± 2.4	0.007	2.2 ± 2.4	2.6 ± 3.5	0.60
V0	-3.1 ± 65.1	-1.7 ± 38.3	0.9			
SV_forward_, mL	54.4 ± 20.0	58.1 ± 18.8	0.42	55.8 ± 17.8	58.0 ± 20.9	0.70
LVEF, %	39.2 ± 13.2	36.3 ± 11.3	0.17	35.6 ± 11.9	36.8 ± 11.8	0.64
LVEF_forward_, %	38.9 ± 21.9	53.4 ± 29.8	<0.001	48.3 ± 27.5	44.0 ± 30.5	0.46
TAPSE, mm	18.1 ± 5.5	15.5 ± 5.8	0.015	15.5 ± 5.8	15.3 ± 5.6	0.86
PASP, mmHg	50.5 ± 16.5	35.7 ± 10.8	<0.001	35.4 ± 10.5	34.4 ± 13.0	0.74
TR Vmax, m/s	3.4 ± 0.6	3.0 ± 0.4	0.017	3.0 ± 0.4	3.0 ± 0.4	0.90
RV-PA coupling, mm/mmHg	0.41 ± 0.21	0.47 ± 0.20	0.19	0.49 ± 0.2	0.65 ± 0.98	0.48
RVFAC, %	31.8 ± 5.6	34.0 ± 5.1	0.22	34.0 ± 5.2	30.7 ± 8.1	0.027

Values are presented as mean ± standard deviation or mean (IQR).

ß indicates stiffness coefficient.

Abbreviations: bpm, beats per minute; e`, early diastolic tissue velocity; E, early mitral inflow velocity; Ea, effective arterial elastance; EDP, end-diastolic pressure; EDV, end-diastolic volume; Ees, end-systolic elastance; ESP, end-systolic pressure; ESV, end-systolic volume; LVEF, left ventricular ejection fraction; LVEF_forward_, forward left ventricular ejection fraction; PASP, pulmonary arterial systolic pressure; RVFAC, right ventricular fractional area change; RV-PA coupling, right ventricular-pulmonary artery coupling; SV_forward_, forward stroke volume; TAPSE, tricuspid annular plane systolic excursion; TMVR, Transcatheter mitral valve replacement; TR, tricuspid regurgitation; V0, calculated ESV at an ESP of 0 mmHg; Vmax, maximum flow velocity; VPed20, calculated EDV at an EDP of 20 mmHg; VPes120, calculated ESV at an ESP of 120 mmHg.

### Right Ventricular Hemodynamic Changes

PASP significantly decreased between baseline and discharge (50.5 ± 16.5 mmHg vs. 35.7 ± 10.8 mmHg, *p* < 0.001) which was maintained from discharge to 1-year follow-up (35.4 ± 10.5 mmHg vs. 34.4 ± 13.0 mmHg, *p* = 0.74). Similarly, TAPSE decreased significantly from baseline to discharge (18.1 ± 5.5 mm vs. 15.5 ± 5.8 mm, *p* = 0.015) with no further changes at follow-up (15.5 ± 5.8 mm vs. 15.3 ± 5.6, *p* = 0.86). Consequently, RV-PA coupling remained stable from baseline to discharge (0.41 ± 0.21 mm/mmHg vs. 0.47 ± 0.20 mm/mmHg, *p* = 0.19) and from discharge to 1-year follow-up (0.49 ± 0.2 mm/mmHg vs. 0.65 ± 0.98 mm/mmHg, *p* = 0.48). While RVFAC did not change significantly between baseline and discharge (31.8% ± 5.6% vs. 34.0% ± 5.1%, *p* = 0.22), a significant decrease in RVFAC between discharge and 1-year follow-up was observed (34.0% ± 5.2% vs. 30.7% ± 8.1%, *p* = 0.027).

### Cox Regression

To assess the prognostic impact of hemodynamic changes following TMVR (measured by VPed20), a Cox regression analysis adjusting for EuroSCORE II was performed. The reduction of VPed20 from baseline to discharge was inversely associated with all-cause mortality or HF hospitalization at 1 year (hazard ratio 0.16, 95% CI 0.04-0.71, *p* = 0.016) as well as with cardiovascular mortality or HF hospitalization at 1 year (hazard ratio 0.18, 95% CI 0.04-0.77, *p* = 0.021).

## Discussion

This is the first study to analyze hemodynamic changes after TMVR using noninvasive pressure-volume loops. In 26 patients with MR treated with dedicated TMVR devices, successful MR elimination resulted in the following changes: reduced end-diastolic and end-systolic LV volumes at discharge, improved LV contractility, improved LVEF_forward_, improved energetic efficiency (conceptually depicted by Ea/Ees ratio), increased EDP, and preserved RV performance (Graphical Abstract). In patients with available 1-year follow-up data, LV volumes increased, but LV contractility and EDP remained stable. Reduction of VPed20 at discharge was associated with beneficial clinical outcomes at 1 year.

### LV Reverse Remodeling After TEER

Currently, available data on the immediate hemodynamic impact of TMVR are scarce. Some prior studies have reported intraprocedural and early short-term postprocedural changes after TMVR,^11^^,^^12^ whereas several studies have investigated the acute hemodynamic and volumetric changes of LV function following TEER addressing cardiac dimensions and parameters of LV contractility.^27^, ^28^, ^29^ Evidence suggests that MR reduction by TEER leads to early LV reverse remodeling with acute reductions in end-systolic and end-diastolic volumes.^17^ Additionally, other studies have demonstrated that a sustainable reduction in LV volumes can be achieved in case of durable resolution of MR. Even in patients with only mild residual MR at 12 months after TEER, the LV volume reduction was still significant.^30^^,^^31^ In contrast, the COAPT trial could not document a sustained reduction in LV volumes after TEER. Nevertheless, the progression of LV remodeling was mitigated after TEER compared to the medical control group.^28^ In a study by Schrage et al. that utilized a similar noninvasive approach as the present study, patients undergoing TEER showed postprocedural improvement in hemodynamic parameters such as VPed20. However, this beneficial impact was only observed for patients with preserved LVEF.^17^

### Left Ventricular Changes After TMVR

In line with previous TMVR studies, a consistent reduction of MR to ≤1+ in all patients resulting in LV reverse remodeling with a reduction of both EDV and ESV was noted in this study.^32^, ^33^, ^34^ A similar impact of TMVR on LV remodeling at 1-month follow-up has been described by Fukui et al.^35^ using cardiac computed tomography scans. In terms of EDV, these changes seem to occur only as acute postprocedural changes detectable at discharge, whereas this effect was reversed in patients with a 1-year follow-up. Of note, when comparing the hemodynamic impact at discharge and follow-up, an incomplete 1-year follow-up must be taken into consideration. In our patient cohort, the decrease in LV volumes immediately after TMVR was paralleled by a leftward shift of the LV pressure-volume relationship, which was valid for both ESPVR and EDPVR, respectively. This was documented by a significant overall decrease in the VPed20 and VPes120. While VPed20 reduction hints at the restoration of hemodynamic LV diastolic function, a decrease in VPes120 is considered to correlate with improvements in LV contractility. This is confirmed by an increase in LVEF_forward_ and, more importantly, by an increase in the Ees slope after TMVR. In addition, we observed energetically more efficient LV performance after TMVR, as indicated by a reduced Ea/Ees ratio. It could be shown that the ratio of LV stroke work and potential energy were optimized at Ea/Ees ratios between 0.3 and 1.3, which corresponds to enhanced Ea/Ees ratios in our study cohort indicating an improvement of ventriculo-arterial coupling.^24^^,^^25^

As mentioned before, the results show an apparent increase in contractility despite lower LV preload, which does not seem consistent with Frank-Starling Law. As per Frank-Starling Law, contractility increases with preload up to a certain point referred to as the descending limb.^36^ Better contractility in the light of less preload could mean that (1) the ventricles operated on the descending limb before TMVR and were optimized after, or (2) more complex LV geometry changes caused by the intervention lead to a better contractile function, resulting in a different Frank-Starling curve then.

Despite improvements in hemodynamic parameters, we observed a nonsignificant decrease of global LVEF. This is a well-known phenomenon following MR treatment, which has been described for both TEER and TMVR^28^^,^^30^^,^^32^^,^^37^ and may be explained by overestimation of LVEF in the presence of MR^38^ as well as some degree of afterload mismatch after eradication of the regurgitant volume following a sudden increase of end-diastolic filling pressures.^39^ Dupuis et al.^40^ were able to show that LVEF_forward_ is superior to global LVEF in the assessment of postoperative LV systolic function in patients undergoing mitral valve surgery. Our study confirms increased filling pressures by MR resolution with an increased EDP following TMVR. Increased LVEF_forward_ further illustrates the fact that proportionally more blood ejection occurs toward the high-pressure circulatory system, leading to increased afterload. Nevertheless, complete restoration of LV function, including diastolic function, would include normalization of EDP, which we could not observe in 1-year follow-up data. This finding requires confirmation by larger prospective studies.

The early hemodynamic changes described in this study are unique to TMVR and have not been reported in similar studies including TEER patients. Complete elimination of MR, as opposed to some degree of residual MR, could be a plausible explanation for this finding and may represent an advantage of TMVR over TEER with potentially prognostic implications.

In general, the beneficial impact of LV reverse remodeling on outcomes after MR treatment has been well described.^30^^,^^41^^,^^42^ Although the sample size of our study was small, our data suggest a significant prognostic impact of early VPed20 reduction on clinical outcomes. Therefore, early LV reverse remodeling could have prognostic implications despite attenuation at follow-up.

### Right Ventricular Changes After TMVR

The impact of TMVR on PA pressures has been consistently described by several studies involving TMVR devices.^33^^,^^37^ Severe MR is frequently associated with some degree of postcapillary pulmonary hypertension, which results from the backward transmission of increased left atrial pressures.^43^ Hence, by eliminating MR, TMVR generally results in effective recovery of PA pressures. Our study supports previous findings that TMVR significantly reduces PASP. However, in our patient population, the decrease in PASP was accompanied by a decline in TAPSE, which might indicate a decline in RV contractility. In line with an early decrease in TAPSE, we observed a significant decrease in RVFAC from discharge to 1-year follow-up. Reduced RV function following mitral valve intervention might be attributed to geometric changes in RV contraction, which may result from the disruption of pericardial integrity following transapical access.^44^ Recently, RV-PA coupling (TAPSE/PASP ratio) has been introduced as an excellent prognostic marker for assessing RV function in patients undergoing TEER.^45^, ^46^, ^47^ Balancing the reduction of both PASP and TAPSE, RV-PA coupling remained stable after TMVR. Given the fact that TAPSE should generally be interpreted in the light of PASP, RV-PA coupling is considered superior to TAPSE alone for defining hemodynamic RV function. Therefore, we conclude that RV function after TMVR was indeed preserved. Nevertheless, the absence of recovery of TAPSE and a decrease in RVFAC at follow-up warrant further investigation of RV performance following TMVR.

### Study Limitations

This study has several limitations that should be acknowledged. First, the absence of a medical or TEER control group limits our ability to assess the natural course of the disease and draw conclusions regarding differences compared to TEER. Second, the retrospective nature of this study warrants verification of the results in prospective studies. Third, the accuracy of echocardiography measurements partly relies on approximation formulas, where small measurement differences may have a significant impact on the overall data. Especially, parameters calculated with measurements regarding the LV outflow tract are typically error prone. Additionally, assessment of both diastolic and systolic function is flawed when considering valve replacement and transapical access. In particular, the assessment of ejection time in the presence (baseline) or absence of MR (following TMVR) remains a potential limitation of the noninvasive approach used in this study. Furthermore, SV_forward_ was used for calculation of Ees at baseline and discharge, thus not considering additional ejection to the left atrium at baseline.

However, we are convinced of the reliability of our results given the somewhat expected hemodynamic changes following MR elimination as demonstrated by noninvasive measurements.^31^ Nevertheless, these results should be validated by studies utilizing invasive hemodynamic measurements. Further studies including a larger patient population with longer echocardiographic follow-up time, are necessary to assess the durability of the short-term hemodynamic and volumetric changes we observed.

## Conclusion

In this single-center study, noninvasive assessment of pressure-volume loops before and after TMVR demonstrated acute LV reverse remodeling and improved LV contractility, while RV performance was preserved. At 1-year follow-up, the LV reverse remodeling was attenuated, but LV contractility remained improved. Considering available data with other treatment options, these findings suggest that complete MR elimination by TMVR may have a greater impact on LV function compared to other MR therapies. In addition, complete abolishment of MR may still be compensated by the ventricle even in the presence of LV dysfunction, irrespective of transapical access. However, it remains to be determined whether the potential benefit of complete MR elimination by TMVR on LV function and reverse remodeling can translate into favorable long-term functional and clinical outcomes.

## Impact on Daily Practice

This study is the first to assess the hemodynamic changes in patients undergoing TMVR by deriving noninvasive assessment of pressure-volume loops. Our results highlight the potential beneficial impact of complete MR elimination on LV contractility and support the need for further investigation of hemodynamics after TMVR. With the transition from transapical to transfemoral/transseptal access in TMVR, procedural safety is likely to improve significantly. As procedural risk improves and TMVR becomes available to a broader patient population, the distinct LV and RV hemodynamic consequences of complete MR elimination and TMVR become more important.

## Ethics Statement

The study was approved by the local ethics committee and was conducted in accordance with the Declaration of Helsinki. Every patient gave written informed consent to the use of data records for scientific research.

## Funding

This study was supported by a personal grant from the 10.13039/501100005971German Heart Foundation (DHS) to S. Ludwig.

## Review Statement

The review of this paper was managed by Guest Editor Fabien Praz, MD.

## Disclosure Statement

S. Ludwig has received travel compensation from Edwards Lifesciences, speaker honoraria from Abbott, and advisory fees from Bayer, and is a consultant to New Valve Technology (NVT). D. Kalbacher has received personal fees from Abbott Medical, Edwards Lifesciences, Medtronic Inc, and Pi-Cardio Ltd. A. Schaefer received speaker honoraria from Abbott. The other authors had no conflicts to declare.
