# Supplementary material for: Left and Right Ventricular Hemodynamic Response After Transcatheter Mitral Valve Replacement
Source: Struct Heart. 2024 Jun 8;8(4):100322. doi: 10.1016/j.shj.2024.100322 (PMC11294893; doi:10.1016/j.shj.2024.100322)
Supplement: Supplementary Material [file mmc1.docx]

**Supplementary Material**

**Supplementary Figure 1.**


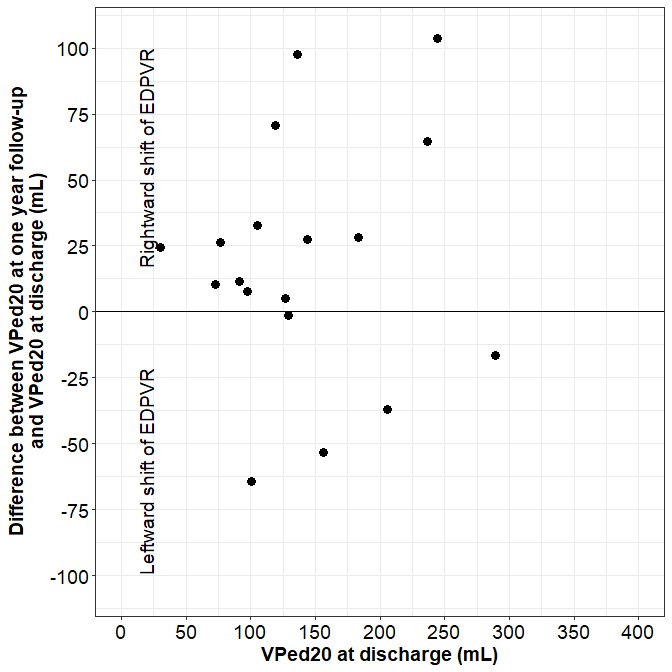


**Supplementary Figure 1.** *End-diastolic pressure-volume relationship between discharge and 1-year follow-up following TMVR.*

Patients are compared based on their calculated end-diastolic volume at an end-diastolic pressure of 20 mm Hg (VPed20) as a marker of the end-diastolic pressure-volume relationship (EDPVR). The y axis displays the change in VPed20 from discharge to 1-year follow-up, and the x axis displays the value of the VPed20 at discharge.
